# MicroRNA-506 modulates insulin resistance in human adipocytes by targeting S6K1 and altering the IRS1/PI3K/AKT insulin signaling pathway

**DOI:** 10.1007/s10863-021-09923-2

**Published:** 2021-10-31

**Authors:** Feng-Yu Zhong, Jing Li, Yu-Mei Wang, Yao Chen, Jia Song, Zi Yang, Lin Zhang, Tian Tian, You-Fang Hu, Zhen-Ying Qin

**Affiliations:** 1 The First Clinical Medical College of Nanjing Medical University, Nanjing, 210029 Jiangsu China; 2 Department of Children’s Health Care, The First Affiliated Hospital of Nanjing Medical University, Jiangsu Maternal and Child Health Care Hospital, Nanjing, 210036 Jiangsu China; 3 Department of Screening for Neonatal Diseases, Huai’an Maternity and Child Health Care Hospital Affiliated to Yangzhou University Medical College, Huaian, 223002 Jiangsu China

**Keywords:** miR-506-3p, S6K1, IRS-1/PI3K/AKT signaling pathway, Insulin resistance, Obesity

## Abstract

The incidence of obesity has increased rapidly, becoming a worldwide public health issue that involves insulin resistance. A growing number of recent studies have demonstrated that microRNAs play a significant role in controlling the insulin signaling network. For example, miR-506-3p expression has been demonstrated to correlate with insulin sensitivity; however, the underlying mechanism remains unknown. In this study, we found that miR-506-3p enhanced glucose uptake by 2-deoxy-D-glucose uptake assays and regulated the protein expression of key genes involved in the PI3K/AKT insulin signaling pathway including IRS1, PI3K, AKT, and GlUT4. We next predicted ribosomal protein S6 kinase B1 (S6K1) to be a candidate target of miR-506-3p by bioinformatics analysis and confirmed using dual-luciferase assays that miR-506-3p regulated S6K1 expression by binding to its 3′-UTR. Moreover, modulating S6K1 expression counteracted the effects of miR-506-3p on glucose uptake and PI3K/AKT pathway activation. In conclusion, miR-506-3p altered IR in adipocytes by regulating S6K1-mediated PI3K/AKT pathway activation. Taken together, these findings provide novel insights and potential targets for IR therapy.

## Introduction

The rate of obesity has increased dramatically worldwide, recently becoming a dominant public health issue (Peters et al. 2018; Reilly 2017). Additionally, there is sufficient evidence to conclude that obesity is a risk factor for type 2 diabetes mellitus (T2DM) (Ghaben and Scherer 2019). T2DM is a common metabolic disease that can cause multiple complications in diverse organs (Wu et al. 2018), these are divided into acute and chronic complications and both lead to poor quality of life and high mortality rates among diabetics. T2DM has been investigated in many ways including proteomics, metabolomics, and metagenomics (Wang et al. 2018), all of which have indicated that obesity-induced insulin resistance (IR) is the primary cause of T2DM (Marchesini et al. 2008; Mozaffarian 2016). IR is defined as decreased glucose availability due to the ineffectiveness of insulin, which leads to decompensation (Kabadi 2017; Petersen and Shulman 2018). The mechanism underlying IR development appears to involve altered transcription of various genes in a synergetic process (Rahimi et al. 2019; Yaribeygi et al. 2019). As a critical characteristic of metabolic syndrome, impaired glucose uptake by adipocytes is closely related to IR (Kabadi 2017; Stern et al. 2016); however, the concrete molecular mechanisms of IR need further exploration.

One of the most important downstream pathways of insulin signaling, the PI3K/AKT pathway plays a central role in regulating glucose and lipid metabolism and is often used to elucidate the hypoglycemic mechanisms of different antidiabetic drugs (Deng et al. 2018). When insulin binds to its homologous receptors on the cell membrane, insulin receptors recruit and phosphorylate intracellular proteins on tyrosine residues, including insulin receptor substrate 1 (IRS1). Phosphorylated IRS1 activates PI3K, which specifically catalyzes the phosphorylation of phosphatidylinositol at the third position of an inositol ring, further activating AKT. AKT is a serine/threonine protein kinase that phosphorylates many other kinases and transcription factors to broadly regulate and participate in glycogen synthesis, fat synthesis, protein synthesis, cell survival, proliferation, and metabolism (Huang et al. 2018). For example, AKT controls glucose and lipid metabolism by activating GLUT4, which is the most important glucose transporter in skeletal muscle and adipocytes (Schultze et al. 2012). Therefore, dysfunction of the PI3K/AKT/GLUT4 axis can lead to abnormal glucose metabolism and IR, which can lead to obesity and T2DM.

S6K1 is serine kinase that contributes to the negative feedback loop that controls insulin activity, especially signaling downstream of AKT in the insulin network (Arif et al. 2017; Bonucci et al. 2020; Moreno-Navarrete et al. 2013; Shum et al. 2016; Sung Hee Um et al. 2004; Um et al. 2004). Under nutrient overload circumstances related to the obese state, S6K1 is phosphorylated at Thr-389, leading to IRS-1 phosphorylation at multiple serine residues, which impairs insulin-stimulated glucose uptake by adipocytes (Um et al. 2006).

MicroRNAs (miRNAs) are endogenous, small, noncoding RNAs of 18 to 24 nucleotides in length that control gene expression by post-transcriptionally inhibiting mRNA translation and increasing mRNA destabilization (Bushati and Cohen 2007; Krol et al. 2010). A previous study (Ludwig et al. 2016) revealed that certain tissues predominantly express specific miRNAs, which account for 17% of miRNAs and miRNA families. These results indicated the potential role of miRNAs in tissue development and for the treatment of tissue-specific diseases. As miRNAs participate in multiple critical signaling pathways, they have been put forward as potential regulators associated with obesity-induced IR and T2DM (Peng and Wang 2018; Ying et al. 2017). In our previous work, we used an miRNA microarray, which indicated that miR-506-3p showed low expression in omental adipose tissue from obese patients. Although miR-506-3p has unknown functions in insulin sensitivity, it has also been reported to be associated with colorectal cancer (Ai et al. 2021), primary sclerosing cholangitis (Kempinska-Podhorodecka et al. 2021), and non-small cell lung cancer (Wang et al. 2021). As indicated in a previous study (Yang et al. 2013), miR-506-3p regulates epithelial-to-mesenchymal transition and DNA damage response. When miR-506-3p was delivered by liposomes, it induced significant tumor regression, suggesting promising clinical potential for miR-506-3p. However, the effects of miR-506-3p on IR are unknown.

In this study, we investigated the function of miR-506-3p in adipocytes and determined that the miR-506-3p/S6K1/PI3K axis regulates glucose uptake in adipocytes. Hence, this study suggests miR-506-3p and its downstream targets could be candidates for treating IR in obesity and T2DM.

## Materials and methods

### Bioinformatics analysis

The PubMed (https://www.ncbi.nlm.nih.gov/pubmed/) and miRBase (http://www.mirbase.org/) (Kozomara et al. 2019) databases were used to identify the sequence of miR-506-3p. According to previous reports, microT-CDS (Maragkakis et al. 2009) and TargetScan (Lewis et al. 2005) are credible tools for predicting the target genes of miRNAs. We selected the common target genes in the hopes of increasing the reliability of the predictions. A Venn diagram was constructed using Bioinformatics & Evolutionary Genomics (http://bioinformatics.psb.ugent.be/). Furthermore, we explored the gene ontology (GO) and Kyoto encyclopedia of genes and genomes (KEGG) for the indicated genes using DAVID Bioinformatics Resources (https://david-bioinformatics.freeforums.net/) (Cun and Yang 2018), which is a database for annotation, visualization, and combined discovery. Additionally, enrichment of GO terms and dot bubbles were performed on http://www.bioinformatics.com.cn, an online platform for analyzing and visualizing data.

### Cell culture and transfection

Human preadipocytes were purchased from ScienCell Research Laboratories (Carlsbad, CA, USA) and maintained in preadipocyte medium (PAM; ScienCell Research Laboratories) containing 5% fetal bovine serum, 1% preadipocyte increase supplement, and 1% penicillin/streptomycin solution at 37 °C in 5% CO_2_. To induce differentiation, confluent adipocyte (day 0) were cultured in Dulbecco’s Modified Eagle Media (DMEM)/F12 (ScienCell Research Laboratories) supplemented with 5 μg/mL insulin (Sigma-Aldrich, St. Louis, MO, USA), 1 μM dexamethasone (Sigma-Aldrich), 0.5 mM 3-isobutyl-1-methylxanthine (Sigma-Aldrich), and 1 μM rosiglitazone (Sigma-Aldrich) for 4 days. Subsequently, the medium was replaced with DMEM/F12 containing 5 μg/mL insulin, which was replaced every 3 days until the appearance of mature lipid droplets (day 15). To evaluate the impact of miR-506-3p on adipocyte IR, adipocytes cultured in PAM were transfected with miR-506-3p mimic, sponge, and negative controls (NCs) including mimic control (Mi-ctrl) and sponge control, all of which were purchased from Genomeditech (Shanghai, China). Full-length *RPS6KB1* (gene encoding S6K1) was cloned into the pcDNA vector (Genomeditech), with empty pcDNA vector serving as the corresponding NC. For transfections, cells were transfected with different miRNAs or plasmids using polybrene (Genomeditech) or the Lipofectamine 2000 kit (Invitrogen, Waltham, MA, USA) based on the manufacturers’ instructions. Cells were collected 24 h after transfection.

### Glucose uptake assay

Stably transfected adipocytes were cultured in 12-well plates and divided into mature adipocytes. Subsequently, mature cells were serum-starved in DMEM/F12 for 48 h to eliminate the effects of differentiation medium, washed twice with KRPH buffer (20 mmol/L HEPES [pH 7.4], 1 mmol/L CaCl_2_, 4.7 mmol/L KCl, 5 mmol/L Na_2_HPO4·12H_2_O, 1 mmol/L MgSO4·7H_2_O, and 1 mmol/L 0.1% bovine serum albumin [BSA]), and then incubated in KRPH buffer with/out 1200 nmol/L insulin for 30 min at room temperature (25 ℃). Then cells were washed with PBS and incubated with labeled 2-deoxy-D-glucose (2-DG; 5 μCi/mL) in 400 μL KRPH buffer for 10 min at 37 °C. The reaction was terminated with ice-cold (4 °C) PBS supplemented with 10 mmol/L D-glucose. Cells were solubilized in 200 μL of 1 mol/L NaOH, and aqueous supernatants of the lysates were transferred to liquid scintillation counters to measure radioactivity. The remainder was applied to bicinchonic acid protein assays (Thermo Fisher Scientific, Waltham, MA, USA) to measure protein concentrations, which were used to normalize radioactivity values. Assays were performed according to protocols published by Ceddia et al. (Ceddia et al. 2005) with minor modifications.

### Luciferase reporter assays

Segments of the *RPS6KB1* 3′-UTR containing putative miR-506-3p bindings sites were synthesized by Genomeditech, and then cleaved with Sac I/Xho I and ligated into the pGL3 plasmid (Promega, Madison, WI, USA) to form the wild-type vector (WT-S6K1). The mutant receptor vector (Mut-S6K1) was formed using a site-directed mutagenesis kit (Stratagene, San Diego, CA, USA) according to the product manual. Then, HEK293T cells (Stem Cell Bank, Chinese Academy of Science, Shanghai, China) were used to validate the miRNA target. HEK293T cells were cultivated in 24-well plates and allowed to grow until reaching approximately 80% confluence. Then they were co-transfected with WT-S6K1 or Mut-S6K1 and miR-506-3p mimic, sponge or NCs using Lipofectamine 2000 kit. At 48 h post-transfection, the Dual Luciferase Assay System (Promega) was used to measure luciferase activities. Renilla was used as a normalization control, and assays were performed in triplicate.

### Western blot analysis

Stably transfected cells were induced to mature adipocytes. After cells had been cultured in serum-free medium for 48 h, they were incubated with DMEM/F12 containing 100 nmol/L insulin for 30 min. Total and phosphorylated proteins were extracted following the instructions provided by the manufacturer of the protein isolation kits (Beyotime, Shanghai, China). Plasma membrane proteins were extracted using Eukaryotic Membrane Protein Extraction Reagent (Pierce, Rockford, IL, USA). Protein concentrations were measured using the bicinchonic acid protein assay kit (Thermo Fisher Scientific) according to the manufacturer’s instructions. For western blotting, 20 μg of protein was loaded in each lane, and after being separated by 8% sodium dodecy1 sulfate–polyacrylamide electrophoresis, proteins were transferred onto polyvinylidene fluoride membranes (Thermo Fisher Scientific). TBS-Tween-20 containing 5% BSA (Beyotime, Shanghai, China) was applied to block membranes for 1 h at room temperature. Membranes were incubated with the following primary antibodies overnight at 4 °C in antibody diluent (Beyotime, Shanghai, China): anti-β-actin, anti-IRS-1, anti-p-IRS-1 (Ser612), anti-PI3K, anti-AKT, anti-p-AKT (Thr308), anti-S6K1, anti-p-S6K1 (Thr389) (all purchased from Cell Signaling Technology, Danvers, MA, USA), and anti-GLUT4 (Santa Cruz Biotechnology, Dallas, TX, USA). Then, the membranes were incubated with secondary goat anti-rabbit IgG (1:10,000) or goat anti-mouse IgG (1:10,000) antibodies conjugated to horseradish peroxidase at room temperature for 1 h. Immunoreactive bands were exposed and quantified using chemiluminescence detection reagent (PerkinElmer, Waltham, MA, USA) and Image J software (National Institutes of Health,USA), respectively.

### Real-time qPCR analysis

When the cells had grown to 70% confluence, we used TRIzol Reagent (Invitrogen) to extract total RNA from cells according to the manufacturer’s protocol. A NanoDrop 2000 (Thermo Fisher Scientific) was used to measure RNA concentrations. Reverse transcribed cDNA was generated using 1000 ng of total RNA with a commercial kit (TaKaRa, Dalian, China). SYBR Premix Ex Taq RNaseH Plus (TaKaRa) was applied to perform qPCR. The small RNA U6 was used as the internal reference for miRNA measurements. Sequences of the primers used in qPCR assays were synthesized by RiboBio (Guangzhou, China) and are listed in Table 1.

**Table 1:** Sequences of the primers used in qPCR assays

Gene	Forward primer	Reverse primer
miR-506-3p	TAAGGCACCCTTCTGAGTAGA	GCGAGCACAGAATTAATACGAC
U6	TGACACGCAAATTCGTGAAGCGTTC	CCAGTCTCAGGGTCCGAGGTATTC
β-actin	CACTATCGGCAA TGAGCGGTTCC	CAGCACTGTGTTGGCATA GAGGT
S6K1	CGGGACGGCTTTTACCCAG	TTTCTCACAATGTTCCATGCCA

### Statistical analysis

All statistical analyses were conducted using SPSS 22.0 (IBM, Armonk, NY, USA) and Prism 8.0 (GraphPad Software, Inc., San Diego, CA, USA) software. All experiments were conducted in triplicate, and all data are presented as mean ± SEM. The Student’s t-test was used for comparisons between two groups, and one-way ANOVA was used for comparisons between multiple groups. P-values < 0.05 were considered statistically significant.

## Results

### MiR-506-3p mimic increased glucose uptake in mature human adipocytes

**Fig. 1:**
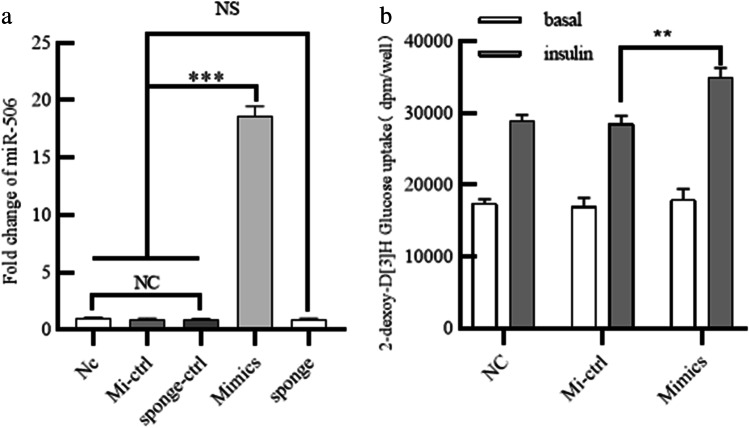
MiR-506-3p mimic increased glucose uptake in mature human adipocytes. Human preadipocytes were transfected with miR-506-3p mimic, sponge, or negative controls (including Mi-ctrl and sponge-ctrl). (a) Transfection efficiency was measured by qPCR. (b) Glucose consumption was evaluated by 2-DG uptake assays. All results are presented as mean ± SEM; *n* = 3; **P < 0.01; ***P < 0.001; NS, no significant difference. NC, negative control; Mi-ctrl: mimic control; sponge-ctrl: sponge control; mimic: miR-506-3p mimic. Datas are presented as fold change compared with NC

To better understand the potential function of miR-506-3p in adipocyte IR, we first determined whether miR-506-3p affected glucose uptake. An overexpression experiment was conducted by transfecting miR-506-3p mimic or NC into adipocytes. Compared with the control group, cells transfected with miR-506-3p mimic had a 20-fold increase in miR-506-3p expression (Fig. 1a), which showed successful transfection of the mimic. Next, the roles of miR-506-3p in IR were studied by conducting 2-DG uptake assays. Compared with the NC groups, glucose uptake of adipocytes was enhanced in the miR-506-3p overexpression group after adding insulin (Fig. 1b). Therefore, miR-506-3p enhanced insulin sensitivity.

### Bioinformatics analysis of miR-506-3p

**Fig. 2:**
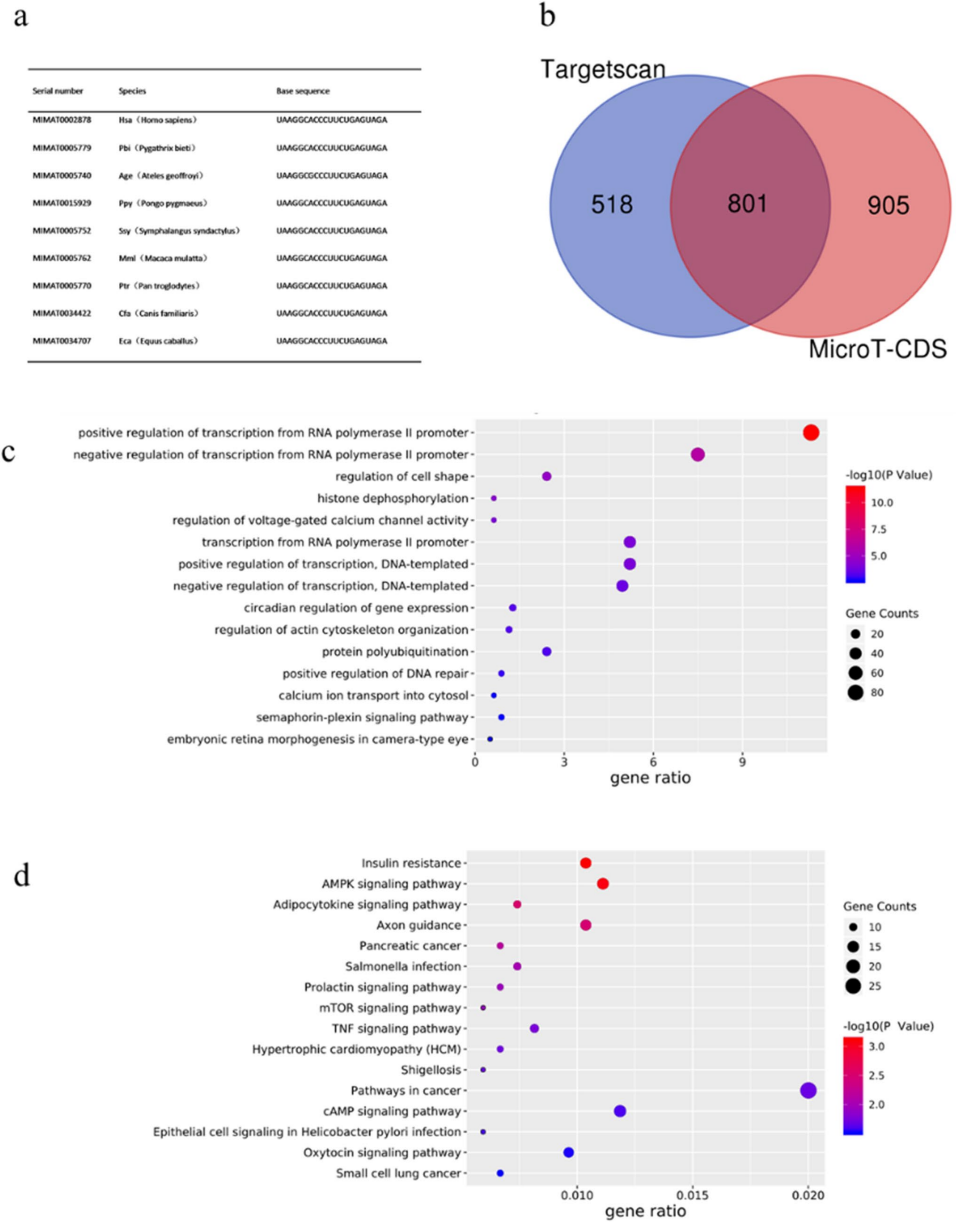
Bioinformatics analysis of miR-506-3p. (a) MiR-506-3p in different species according to the PubMed and miRBase databases. (b) Target genes predicted by the microT-CDS and TargetScan databases. (c) Biological process analysis of hsa-miR-506-3p.(d) KEGG pathway analysis of hsa-miR-506-3p

The sequence of miR-506-3p was compared between species using PubMed and miRBase, which revealed high evolutionary conservation, suggesting vital biological function (Fig. 2a). Target identification is crucial for the functional characterization of miRNAs. According to previous reports, microT-CDS is a reliable tool for predicting target genes of microRNAs; additionally, TargetScan can predict target genes of microRNAs across multiple genomes. The microT-CDS and TargetScan databases were used to predict target genes of miR-506-3p. To increase reliability of the predicted targets, we selected the 801 target genes common to both databases (Fig. 2b). To determine which GO and KEGG pathways were regulated by miR-506-3p, we compared the target genes against GO biological processes and KEGG pathways. Among the GO biological processes, predicted targets were significantly overrepresented in the post-transcriptional level of regulating gene expression (Fig. 2c). KEGG pathway enrichment analysis of the predicted target genes revealed that almost all miR-506-3p targets were involved in the IR, AMPK, and adipocytokine signaling pathways (Fig. 2d). In summary, miR-506-3p is involved in regulating glucose uptake, particularly in IR, by mediating translational inhibition.

### MiR-506-3p directly targets RPS6KB1 (gene encoding S6K1)

**Fig. 3:**
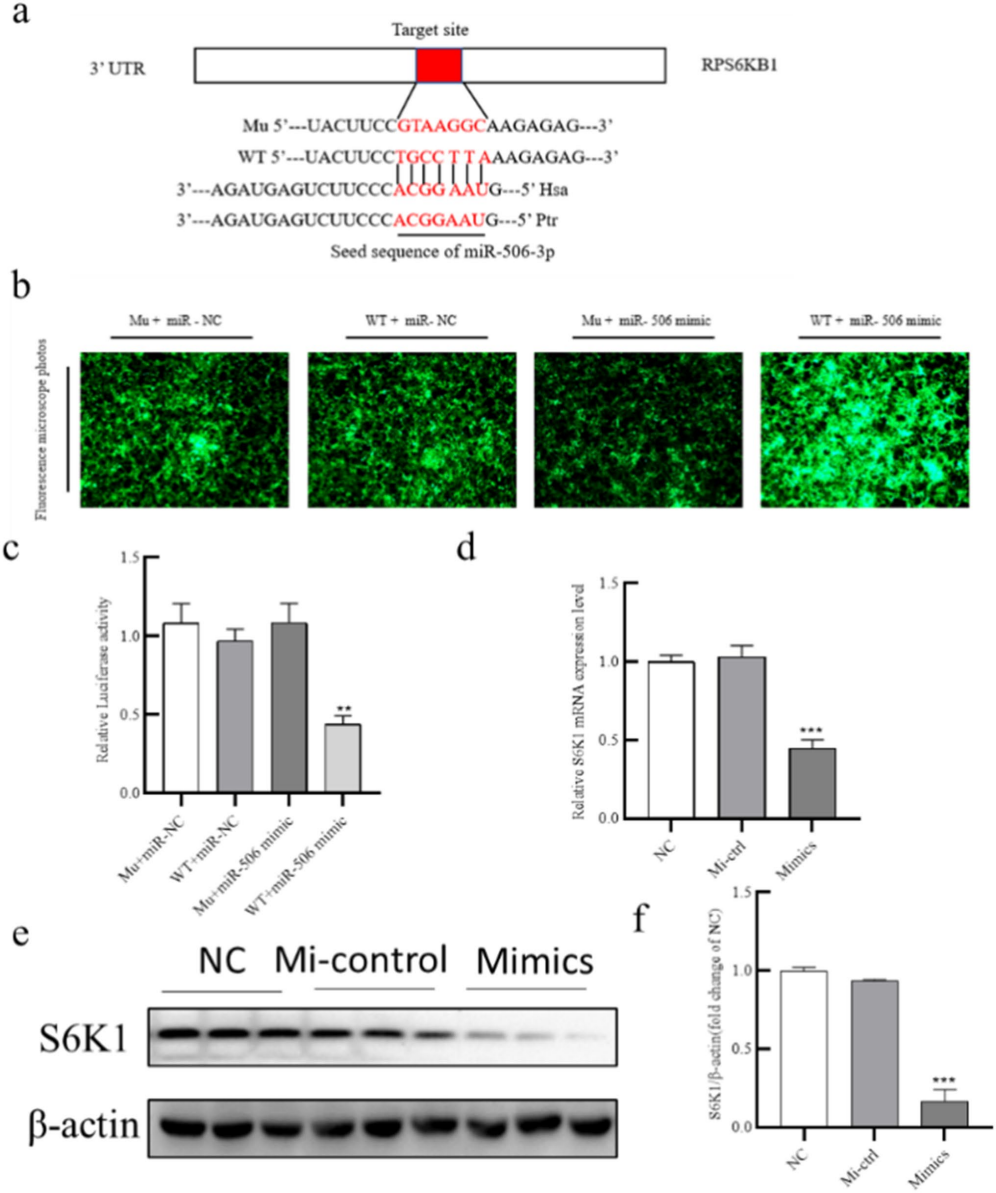
MiR-506-3p directly targets RPS6KB1. (a) Sequence alignment of miR-506-3p with the 3′-UTR of human (Hsa) and chimpanzee (Ptr) *RPS6KB1* mRNA. The binding site and seed region of miR-506-3p are indicated in red. (b) Lentiviral transfection efficiency was measured by fluoroscopy. (c) The effect of miR-506-3p mimic and miR-NC on the activity of WT-S6K1 and Mut-S6K1 were measured by luciferase assays. (d) *RPS6KB1* mRNA levels after transfection with miR-506-3p mimic, NC, and Mi-ctrl were measured by qPCR. (e–f) S6K1 protein levels after transfection with miR-506-3p mimic, NC, and Mi-ctrl were measured by western blot. All results are presented as mean ± SEM; *n* = 3; *P < 0.05; **P < 0.01; ***P < 0.001. NC, negative control; Mi-ctrl: mimic control; mimic: miR-506-3p mimic; WT-S6K1, luciferase plasmid with wild-type *RPS6KB1* 3′-UTR; Mut-S6K1, luciferase plasmid with mutant *RPS6KB1* 3′-UTR

MiRNAs play significant roles in the insulin signaling pathway of adipocytes by binding to complementary sites within the 3′-UTRs of target genes. TargetScan was used to discover the molecular mechanism through which miR-506-3p regulated glucose uptake in adipocytes by predicting miR-506-3p target genes. Among the predicted target genes, we noted that *RPS6KB1* had a complementary sequence to miR-506-3p in its 3′-UTR (Fig. 3a). To test the hypothesis that miR-506-3p directly regulates *RPS6KB1* by binding to its 3′-UTR, we conducted dual-luciferase assays. As shown in Fig. 3c, compared with the miR-NC + WT-S6K1 group, miR-506-3p mimic greatly depressed luciferase activity of WT-S6K1 by binding to the inserted segment of the *RPS6KB1* 3′-UTR. As expected, luciferase activity was unchanged in cells expressing Mut-S6K1 + miR-506-3p mimic or miR-NC (Fig. 3c). To conform these results, qPCR and western blot analysis were conducted to determine the relationship between miR-506-3p and S6K1, respectively. The results indicated that miR-506-3p mimic downregulated the mRNA and protein levels of S6K1 in mature human adipocytes (Fig. 3d–f). Together, these findings indicated that miR-506-3p directly targets *RPS6KB1*.

### MiR-506-3p mimic promoted insulin sensitivity by altering PI3K/AKT activity

**Fig. 4:**
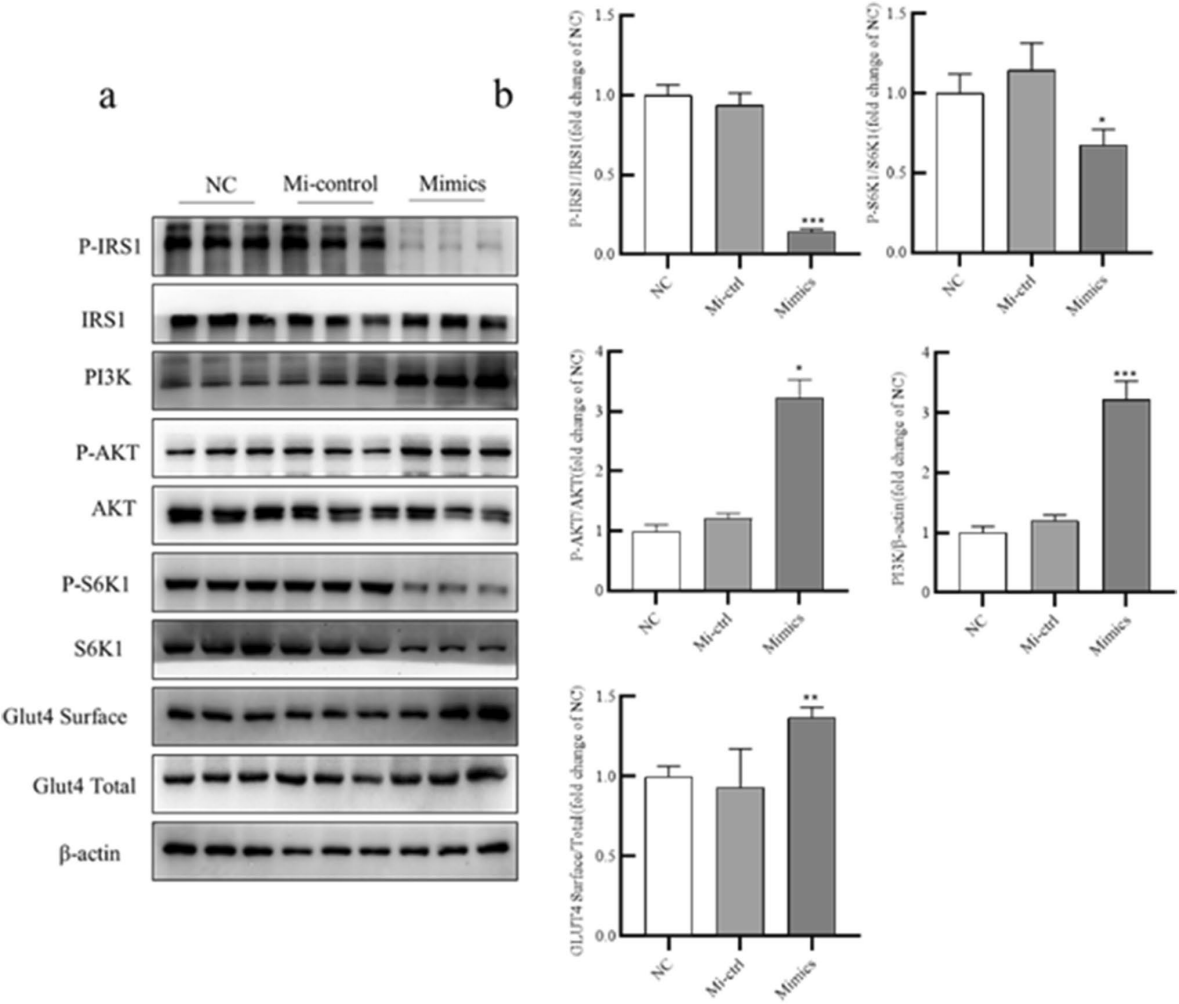
MiR-506-3p mimic promoted insulin sensitivity by altering PI3K/AKT activity. (a) Human preadipocytes were transfected with miR-506-3p mimic, Mi-ctrl or NC, and then western blotting was used to examine the protein levels of phosphorylated P-IRS-1, IRS-1, PI3K, P-AKT, AKT, P-S6K1, S6K1, Glut4 Surface, Glut4 (Total), and β-actin in adipocytes. (b) Quantification of the gray values in (a). All results are presented as mean ± SEM; *n* = 3; *P < 0.05; **P < 0.01; ***P < 0.001 compared with NC. NC, negative control; Mi-ctrl: mimic control; mimic: miR-506-3p mimic

Given the important role of the PI3K/AKT cascade in IR, adipocytes were treated with synthetic miR-506-3p mimic (or NC) to detect whether miR-506-3p regulated the activity of key proteins in the PI3K/AKT pathway. The results revealed that miR-506-3p mimic reduced levels of phosphorylated IRS-1 (Ser612) and S6K1 (Thr389) and increased levels of phosphorylated AKT (Thr308), total PI3K, and of Glut4 surface expression (Fig. 4a, b). Thus, miR-506-3p promotes insulin sensitivity by altering PI3K/AKT signaling.

### Sponging miR-506-3p reduced glucose uptake by mature human adipocytes and increased S6K1 expression

**Fig. 5:**
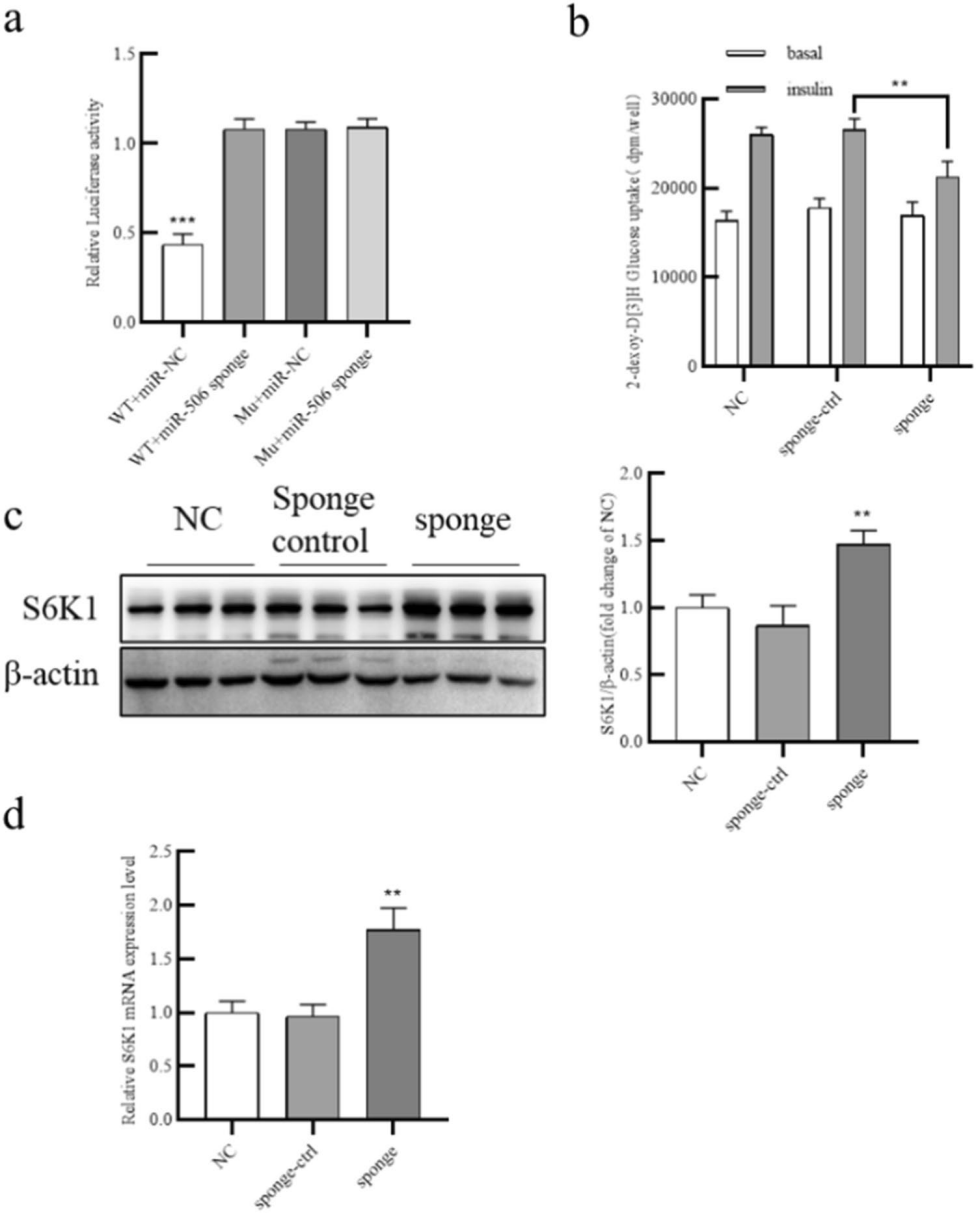
Sponging miR-506-3p reduced glucose uptake by mature human adipocytes. (a) Luciferase activity was detected in adipocytes transfected with WT-S6KB1 or Mut-S6KB1 and miR-506-3p sponge or miR-NC. (a) Human preadipocytes were transfected with miR-506-3p sponge and sponge control, and then glucose consumption was evaluated by 2-DG uptake assays. (c) S6K1 protein expression was analyzed by western blot. (d) *RPS6KB1* mRNA expression was analyzed by qPCR. All results are presented as mean ± SEM; *n* = 3; *P < 0.05; **P < 0.01; ***P < 0.001 compared with NC. NC, negative control; WT-S6K1, luciferase plasmid with wild-type *RPS6KB1* 3′-UTR; Mut-S6K1, luciferase plasmid with mutant *RPS6KB1* 3′-UTR

To further confirm the effect of miR-506-3p on S6K1, we transfected adipocytes with miR-506-3p sponge and sponge control. As shown in Fig. 5a, dual-luciferase reporter assays showed that compared with NC, miR-506-3p sponge depressed the luciferase activity of WT-S6K1 by binding to the inserted segment of the *RPS6KB1* 3′-UTR. Next, 2-DG uptake assays were performed to evaluate the role of miR-506-3p on glucose consumption. After adding insulin, glucose uptake was decreased in adipocytes treated with miR-506-3p sponge compared with the control groups (Fig. 5b). Thus, miR-506-3p promotes insulin sensitivity. To further investigate the relationship between miR-506-3p and *RPS6KB1*, we transfected miR-506-3p sponge into adipocytes and found that the mRNA and protein levels of S6K1 were increased in the miR-506-3p sponge group compared with NC (Fig. 5c, d). In summary, miR-506-3p sponge promoted S6K1 expression and reduced glucose uptake in mature human adipocytes.

### Sponging miR-506-3p impaired insulin sensitivity by altering PI3K/AKT activity

**Fig. 6:**
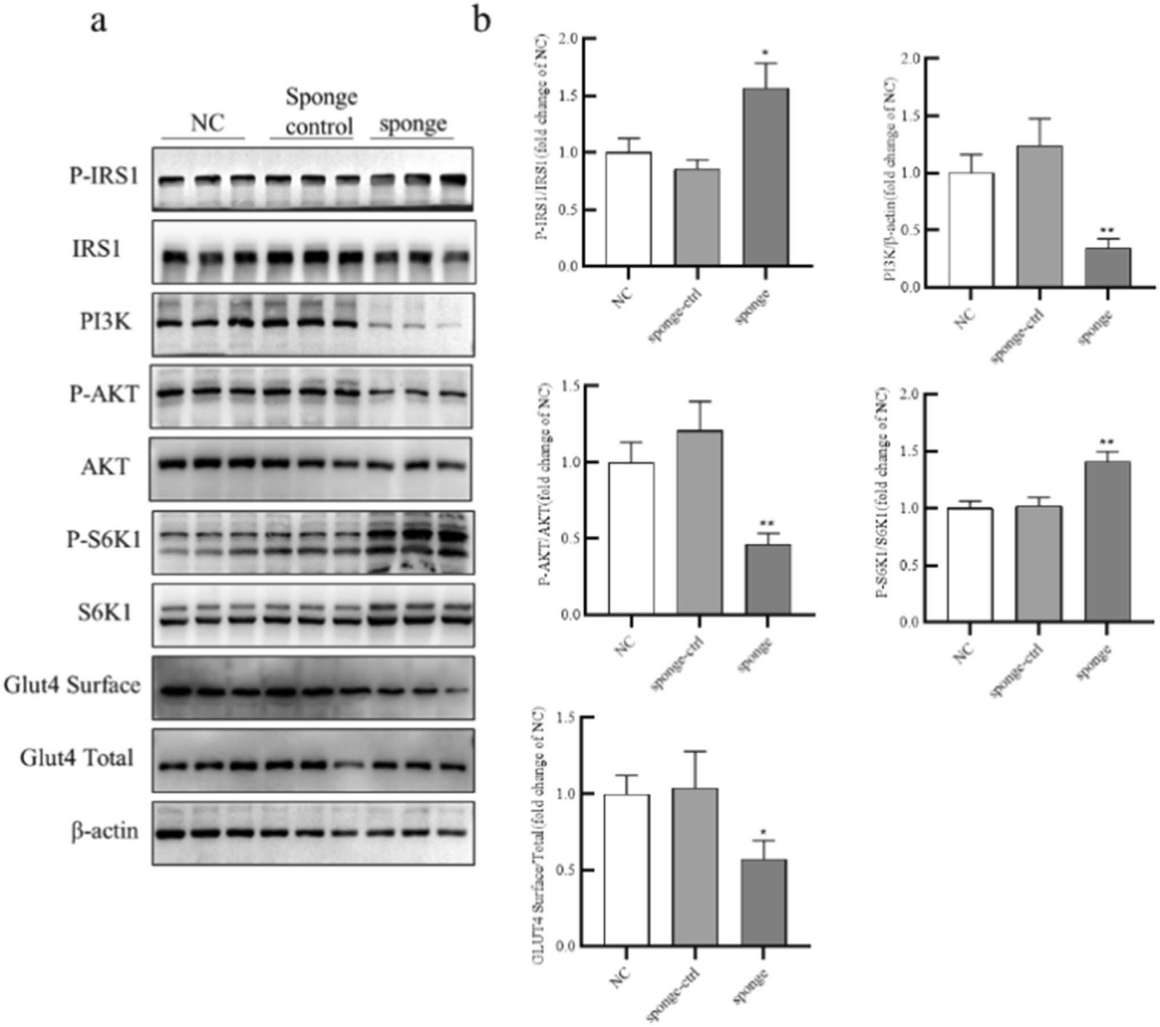
Sponging miR-506-3p impaired insulin sensitivity by altering PI3K/AKT activity. (a) Human preadipocytes were transfected miR-506-3p sponge, sponge control, and NC, and then western blotting was used to measure protein levels of P-IRS-1, IRS-1, PI3K, P-AKT, AKT, P-S6K1, S6K1, Glut4 Surface, Glut4 (Total), and β-actin. (b) Quantification of the gray values in (a). All results are presented as mean ± SEM; *n* = 3; *P < 0.05; **P < 0.01; ***P < 0.001 compared with NC. NC, negative control

To further investigate the relationship between miR-506-3p and the PI3K/AKT pathway, we transfected miR-506-3p sponge and NC in adipocytes. As shown in Fig. 6a and b, miR-506-3p sponge enhanced levels of P-IRS-1 (Ser612) and P-S6K1 (Thr389) and decreased levels of phosphorylated AKT (Thr308), total PI3K, and Glut4 surface expression. Thus, these data confirmed that miR-506-3p promotes insulin sensitivity by altering PI3K/AKT activity.

### Overexpressing S6K1 reversed the effects of miR-506-3p on glucose uptake and PI3K/AKT activity

**Fig. 7:**
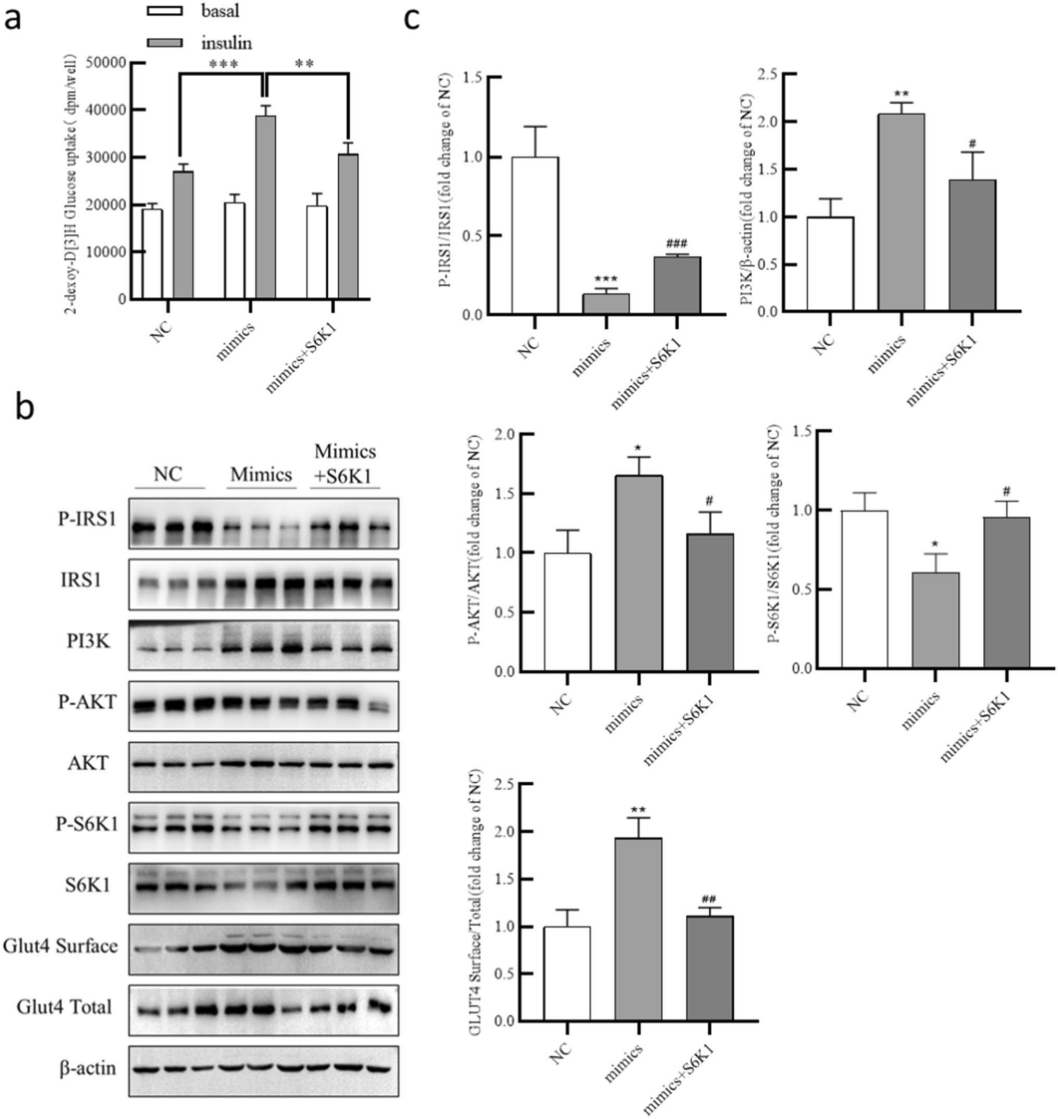
Overexpressing S6K1 counteracted the effects of miR-506-3p mimic on glucose uptake and PI3K/AKT activity. (a) Human preadipocytes were transfected with miR-506-3p mimic, S6K1 overexpression vector, and Mi-ctrl with S6K1 vector control (NC), and then 2-DG uptake assays were performed to evaluate glucose uptake by each group. (b) Western blotting was performed to measure the protein expression of P-IRS-1, IRS-1, PI3K, P-AKT, AKT, P-S6K1, S6K1, Glut4 Surface, Glut4 (Total), and β-actin. (c) Quantification of the gray values in (b). All results are presented as mean ± SEM; *n* = 3; *P < 0.05; **P < 0.01; ***P < 0.001 compared with NC. NC, mimics control (Mi-ctrl) and S6K1 vector control

To further confirm the role of S6K1 in glucose uptake, we overexpressed S6K1 in adipocytes. As shown in Fig. 7a, transfecting miR-506-3p mimic increased glucose consumption compared with the NC group; however, further adding the S6K1 overexpression plasmid reduced glucose consumption. Moreover, western blot results showed that overexpressing S6K1 reversed the suppressive effects of miR-506-3p mimic on the levels of P-IRS-1 (Ser612) and P-S6K1 (Thr389) and on the miR-506-3p-induced increases in P-AKT (Thr308), total PI3K, and Glut4 surface expression (Fig. 7b). Taken together, these data suggest that *S6K1 *can reverse the effects of miR-506-3p on glucose uptake and PI3K/AKT activity.

## Discussion

IR is known to lead to the development of T2DM, and obesity is one of the major underlying causes of IR (Roden and Shulman 2019). The incidence rate of obesity is increasing worldwide, with obesity now affecting more than 33% of the world’s population. Thus, a new method of ameliorating IR is necessary. Some miRNAs are considered fundamental, as they target key genes that participate in IR (Rupaimoole and Slack 2017). In this study, we found that miR-506-3p regulated glucose uptake of adipocytes by controlling the PI3K/AKT insulin signaling pathway, with *RPS6KB1* being a target mRNA of miR-506-3p.

In this study, we first conducted glucose uptake assays to investigate the biological function of miR-506-3p. These data revealed that miR-506-3p expression was positively correlated with glucose uptake levels of adipocytes, revealing that this miRNA may act as a positive regulator of cellular glucose absorption. IR primarily occurs because of decreased biological effects of circulating insulin; meanwhile, the sensitivity of adipose tissue to insulin simultaneously decreases, rendering it unable to absorb and use glucose normally. Thus, we hypothesize that miR-506-3p could ameliorate IR by increasing glucose uptake by adipocytes. Next, we found that miR-506-3p regulated PI3K/AKT activity. Western blot results showed that miR-506-3p regulated levels of P-IRS-1, P-AKT, Glut4 surface expression, and PI3K. The PI3K/AKT/GLUT4 axis in the insulin signaling network is one of the most active metabolic pathways in adipose tissue. In adipose tissue, the combination of insulin with IRS1 activates the inherent tyrosine kinase activity of the receptor, causing autophosphorylation of IRS1. P-IRS1 activates PI3K downstream of the insulin receptor, which ultimately leads to AKT activation through signaling intermediates (Richter and Hargreaves 2013). AKT mediates the glucose uptake signaling pathway and is the key downstream target of the PI3K pathway. AKT can stimulate the glucose uptake by adipose tissue via activating GLUT4 (Richter and Hargreaves 2013). Increase GLUT4 expression improves insulin sensitivity in liver and adipose tissues, reducing blood glucose levels (Huang et al. 2019). The main cause of IR is disruption of the insulin signaling pathway. Zhang et al. found that miR-506-3p alleviated myocardial ischemia–reperfusion injury via targeting PI3K/AKT (Zhang et al. 2020). Similarly, Lin et al. reported that MiR-506-3p inhibited colorectal cancer cell proliferation and invasion by regulating PI3K (Lin et al. 2019). These reports are consistent with our results, and together confirm the connection between miR-506-3p and the PI3K/AKT pathway. Additionally, these findings suggest that miR-506-3p/PI3K/AKT may be an important signaling axis in various other biological processes and diseases.

MiRNAs play important roles in many biological processes by negatively regulating target gene expression. As demonstrated by bioinformatics analysis, regulation of transcription from RNA polymerase II promoters was a main biological process of miR-506-3p, and consistent with classical roles, S6K1 was a feasible target gene. Recent studies have demonstrated that S6K1 participates in IR and obesity. For example, S6K1-deficient mice fed a high-fat diet do not show obesity or diabetes (Sung Hee Um et al. 2004; Um et al. 2006). We found that overexpressing miR-506-3p mimic repressed S6K1 expression, while miR-506-3p sponge increased S6K1 levels. Additionally, dual-luciferase assays confirmed that S6K1 contained a conserved miR-506-3p binding site. Together with previously published data, our results demonstrate that *RPS6KB1* mRNA is a direct target of miR-506-3p.

Overexpressing S6K1 in adipocytes revealed that S6K1 could reverse the elevated glucose uptake and dysregulation of PI3K/AKT signaling induced by miR-506-3p mimic. Previous studies have reported that S6K1 inhibits PI3K/AKT pathway activation and negatively regulates insulin signaling by phosphorylating IRS-1 under excessive nutrient conditions (Copps and White 2012; Sung Hee Um et al. 2004; Um et al. 2006). The study conducted by Arif et al. established a molecular mechanism through which different upstream signaling cascades are integrated through multi-site phosphorylation of S6K1, which in turn dictates substrate selection; these data revealed the multiple and indispensable roles of S6K1 in the PI3K/AKT signaling pathway (Arif et al. 2019). Another study suggested that S6K1 links tumor necrosis factor-α to the negative feedback loop that includes PI3K/AKT-mediated inhibition of insulin signaling by directly phosphorylating IRS-1 on Ser-270 (Zhang et al. 2008). Thus, S6K1 may be the connection between the PI3K/AKT pathway and miR-506-3p.

Our results showed that miR-506-3p plays an important role in IR and obesity. However, this study lacks in vivo experimental results, and whether miR-506-3p has a therapeutic effect on IR requires verification in animal models. Furthermore, future studies are necessary to investigate other potential mechanisms through which miR-506-3p regulates IR, such as transcriptomics.

## Conclusion

In this study, we revealed a novel mechanistic role of miR-506-3p in regulating IR and validated that miR-506-3p directly targets *RPS6KB1* mRNA to regulate the IRS1/PI3K/AKT insulin signaling pathway. These novel findings regarding miR-506-3p activity not only advances our knowledge of miRNA activity in IR but also suggest that miR-506-3p could be identified a promising therapeutic target for treating obesity and T2DM.

## Data Availability

The datasets generated during and/or analysed during the current study are available from the corresponding author on reasonable request.

## Declarations

### Author statement

All authors have seen and approved the final version of the manuscript being submitted. We warrant that the article is the authors’ original work, hasn’t received prior publication and isn’t under consideration for publication elsewhere.

## References

[CR1] Ai L, Luo X, Yan X, Jiang S (2021). MicroRNA-506-3p inhibits colorectal cancer cell proliferation through targeting enhancer of zeste homologue 2. Bioengineered.

[CR2] Arif A, Terenzi F, Potdar AA, Jia J, Sacks J, China A, Halawani D, Vasu K, Li X, Mark Brown J, Chen J, Kozma SC, Thomas G, Fox PL (2017). EPRS is a critical mTORC1-S6K1 effector that influences adiposity in mice. Nature.

[CR3] Arif A, Jia J, Willard B, Li X, Fox PL (2019) Multisite phosphorylation of S6K1 directs a Kinase phospho-code that determines substrate selection. Mol Cell 73:446–457.e446. 10.1016/j.molcel.2018.11.017 10.1016/j.molcel.2018.11.017 PMC6415305 30612880

[CR4] Bonucci M, Kuperwasser N, Barbe S, Koka V, de Villeneuve D, Zhang C, Srivastava N, Jia X, Stokes MP, Bienaimé F, Verkarre V, Baptiste Lopez J, Jaulin F, Pontoglio M, Terzi F, Delaval B, Piel M, Pende M (2020). mTOR and S6K1 drive polycystic kidney by the control of Afadin-dependent oriented cell division. Nat Commun.

[CR5] Bushati N, Cohen SM (2007). microRNA functions. Annu Rev Cell Dev Biol.

[CR6] Ceddia RB, Somwar R, Maida A, Fang X, Bikopoulos G, Sweeney G (2005). Globular adiponectin increases GLUT4 translocation and glucose uptake but reduces glycogen synthesis in rat skeletal muscle cells. Diabetologia.

[CR7] Copps KD, White MF (2012) Regulation of insulin sensitivity by serine/threonine phosphorylation of insulin receptor substrate proteins IRS1 and IRS2. Diabetologia 55:2565–2582. 10.1007/s00125-012-2644-8 10.1007/s00125-012-2644-8 PMC4011499 22869320

[CR8] Cun J, Yang Q (2018). Bioinformatics-based interaction analysis of miR-92a-3p and key genes in tamoxifen-resistant breast cancer cells. Biomed Pharmacother.

[CR9] Deng T, Zhang Y, Wu Y, Ma P, Duan J, Qin W, Yang X, Chen M (2018). Dibutyl phthalate exposure aggravates type 2 diabetes by disrupting the insulin-mediated PI3K/AKT signaling pathway. Toxicol Lett.

[CR10] Ghaben AL, Scherer PE (2019). Adipogenesis and metabolic health. Nat Rev Mol Cell Biol.

[CR11] Huang X, Liu G, Guo J, Su Z (2018). The PI3K/AKT pathway in obesity and type 2 diabetes. Int J Biol Sci.

[CR12] Huang F, Chen J, Wang J, Zhu P, Lin W (2019). Palmitic Acid induces MicroRNA-221 expression to decrease glucose uptake in HepG2 cells via the PI3K/AKT/GLUT4 pathway. BioMed Res Int.

[CR13] Kabadi UM (2017). Major pathophysiology in prediabetes and type 2 diabetes: decreased insulin in lean and insulin resistance in obese. J Endocr Soc.

[CR14] Kempinska-Podhorodecka A, Adamowicz M, Ostrycharz E, Chmielarz M, Wójcicki M, Milkiewicz P, Milkiewicz M (2021). Role of miR-506-3p in ulcerative colitis associated with primary sclerosing cholangitis. Sci Rep.

[CR15] Kozomara A, Birgaoanu M, Griffiths-Jones S (2019). miRBase: from microRNA sequences to function. Nucleic Acids Res.

[CR16] Krol J, Loedige I, Filipowicz W (2010). The widespread regulation of microRNA biogenesis, function and decay. Nat Rev Genet.

[CR17] Lewis BP, Burge CB, Bartel DP (2005). Conserved seed pairing, often flanked by adenosines, indicates that thousands of human genes are microRNA targets. Cell.

[CR18] Lin Y, Chen Z, Zheng Y, Liu Y, Gao J, Lin S, Chen S (2019) MiR-506 Targets UHRF1 to inhibit colorectal cancer proliferation and invasion via the kiss1/pi3k/nf-κb signaling axis. Front Cell Dev Biol 7:266. 10.3389/fcell.2019.00266 10.3389/fcell.2019.00266 PMC6873823 31803739

[CR19] Ludwig N, Leidinger P, Becker K, Backes C, Fehlmann T, Pallasch C, Rheinheimer S, Meder B, Stähler C, Meese E, Keller A (2016). Distribution of miRNA expression across human tissues. Nucleic Acids Res.

[CR20] Maragkakis M, Reczko M, Simossis VA, Alexiou P, Papadopoulos GL, Dalamagas T, Giannopoulos G, Goumas G, Koukis E, Kourtis K, Vergoulis T, Koziris N, Sellis T, Tsanakas P, Hatzigeorgiou AG (2009). DIANA-microT web server: elucidating microRNA functions through target prediction. Nucleic Acids Res.

[CR21] Marchesini G, Moscatiello S, Di Domizio S, Forlani G (2008). Obesity-associated liver disease. J Clin Endocrinol Metab.

[CR22] Moreno-Navarrete JM, Ortega F, Sánchez-Garrido MA, Sabater M, Ricart W, Zorzano A, Tena-Sempere M, Manuel Fernández-Real J (2013). Phosphorylated S6K1 (Thr389) is a molecular adipose tissue marker of altered glucose tolerance. J Nutr Biochem.

[CR23] Mozaffarian D (2016). Dietary and policy priorities for cardiovascular disease, diabetes, and obesity: a comprehensive review. Circulation.

[CR24] Peng C, Wang Y-L (2018). Editorial: MicroRNAs as New Players in Endocrinology. Front Endocrinol (Lausanne).

[CR25] Peters U, Dixon AE, Forno E (2018). Obesity and asthma. J Allergy Clin Immunol.

[CR26] Petersen MC, Shulman GI (2018). Mechanisms of insulin action and insulin resistance. Physiol Rev.

[CR27] Rahimi E, Ahmadi A, Boroumand MA, Mohammad Soltani B, Behmanesh M (2019). Nutrient sensing pathway genes expression dysregulated in patients with T2DM and coronary artery disease. Diabetes Res Clin Pract.

[CR28] Reilly JJ (2017). Health effects of overweight and obesity in 195 countries. N Engl J Med.

[CR29] Richter EA, Hargreaves M (2013). Exercise, GLUT4, and skeletal muscle glucose uptake. Physiol Rev.

[CR30] Roden M, Shulman GI (2019). The integrative biology of type 2 diabetes. Nature.

[CR31] Rupaimoole R, Slack FJ (2017). MicroRNA therapeutics: towards a new era for the management of cancer and other diseases. Nat Rev Drug Discov.

[CR32] Schultze SM, Hemmings BA, Niessen M, Tschopp O (2012). PI3K/AKT, MAPK and AMPK signalling: protein kinases in glucose homeostasis. Expert Rev Mol Med.

[CR33] Shum M, Bellmann K, St-Pierre P, Marette A (2016). Pharmacological inhibition of S6K1 increases glucose metabolism and Akt signalling in vitro and in diet-induced obese mice. Diabetologia.

[CR34] Stern JH, Rutkowski JM, Scherer PE (2016). Adiponectin, Leptin, and Fatty acids in the maintenance of metabolic homeostasis through adipose tissue crosstalk. Cell Metab.

[CR35] Sung Hee Um FF, Watanabe M, Picard F, Joaquin M, Sticker M, Fumagalli S, Allegrini PR, Kozma SC, Auwerx J, Thomas G (2004). Absence of S6K1 protects against age-and-diet-induced obesity while enhancing insulin sensitivity. Nature.

[CR36] Um SH, Frigerio F, Watanabe M, Picard F, Joaquin M, Sticker M, Fumagalli S, Allegrini PR, Kozma SC, Auwerx J, Thomas G (2004). Absence of S6K1 protects against age- and diet-induced obesity while enhancing insulin sensitivity. Nature.

[CR37] Um SH, D'Alessio D, Thomas G (2006). Nutrient overload, insulin resistance, and ribosomal protein S6 kinase 1, S6K1. Cell Metab.

[CR38] Wang N, Zhu F, Chen L, Chen K (2018). Proteomics, metabolomics and metagenomics for type 2 diabetes and its complications. Life Sci.

[CR39] Wang K, Wang B, Wang Z, Yang R (2021). Alginic acid inhibits non-small cell lung cancer-induced angiogenesis via activating miR-506-3p expression. J Nat Med.

[CR40] Wu T, Qiao S, Shi C, Wang S, Ji G (2018). Metabolomics window into diabetic complications. J Diabetes Investig.

[CR41] Yang D, Sun Y, Hu L (2013). Integrated analyses identify a master microRNA regulatory network for the mesenchymal subtype in serous ovarian cancer. Cancer Cell.

[CR42] Yaribeygi H, Farrokhi FR, Butler AE, Sahebkar A (2019). Insulin resistance: Review of the underlying molecular mechanisms. J Cell Physiol.

[CR43] Ying W, Riopel M, Bandyopadhyay G, Dong Y, Birmingham A, Seo JB, Ofrecio JM, Wollam J, Hernandez-Carretero A, Fu W, Li P, Olefsky JM (2017). Adipose tissue macrophage-derived exosomal mirnas can modulate in vivo and in vitro insulin sensitivity. Cell.

[CR44] Zhang J, Gao Z, Yin J, Quon MJ, Ye J (2008) S6K directly phosphorylates IRS-1 on Ser-270 to promote insulin resistance in response to TNF-(alpha) signaling through IKK2. J Biol Chem 283:35375–35382. 10.1074/jbc.M806480200 10.1074/jbc.M806480200 PMC2602883 18952604

[CR45] Zhang M, Wang JY, Li L, Li GM (2020) MiR-506 alleviates myocardial ischemia-reperfusion injury via targeting PI3K/AKT. Eur Rev Med Pharmacol Sci 24:12896–12903. 10.26355/eurrev_202012_24193 10.26355/eurrev_202012_24193 33378040

